# HIV/AIDS-related hyponatremia: an old but still serious problem

**DOI:** 10.1080/0886022X.2017.1419975

**Published:** 2018-01-04

**Authors:** Zhanjun Shu, Zimeng Tian, Jinglin Chen, Jianping Ma, Aihemaiti Abudureyimu, Qianqian Qian, Li Zhuo

**Affiliations:** a AIDS Research Office, National Traditional Chinese Medicine Research Base in Xinjiang, Urumqi, People’s Republic of China;; b Department of Nephrology, China-Japan Friendship Hospital, Beijing, People’s Republic of China;; c Xinjiang Medical University, Urumuqi, People’s Republic of China;; d Xinjiang Uygur Autonomous Regional the Sixth People’s Hospital, Urumqi, People’s Republic of China

**Keywords:** HIV, AIDS, hyponatremia

## Abstract

Hyponatremia is the most common electrolyte disorder in hospitals. Many medical illnesses, including congestive heart failure, liver failure, renal failure and pneumonia, may be associated with hyponatremia. In addition, hyponatremia in patients with the acquired immunodeficiency syndrome (AIDS) and AIDS-related complex (ARC) was first reported in 1993. The evidence suggests that severe hyponatremia is associated with increased morbidity and mortality in human immunodeficiency virus (HIV)/AIDS patients; however, the incidence of hyponatremic syndrome in HIV/AIDS patients remains very high in clinical practice, as almost 40% of HIV/AIDS inpatients in Xinjiang, a developing region of China, are hyponatremic. A method for identifying the pathogenesis and therapeutic treatments for hyponatremia in HIV/AIDS patients is needed. This review focuses on the clinical and pathophysiological aspects of hyponatremia and highlights the causes, presentation and treatment recommendations for hyponatremic patients with HIV/AIDS.

## 1. Hyponatremia in HIV/AIDS

Hyponatremia is defined as serum sodium levels less than 135 mEq/L. Symptoms of hyponatremia depend on its severity and the rate of sodium decline. Gradual decrease in sodium usually results in minimal symptoms, whereas rapid decrease can result in severe symptoms. Polydipsia, muscle cramps, headaches, falls, confusion, altered mental status, obtundation, coma and status epilepticus may indicate the need for acute intervention. Most patients with hyponatremia are asymptomatic, and hyponatremia is noted incidentally. Overt neurologic symptoms are most often due to very low-serum sodium levels (usually <115 mEq/L), which result in intracerebral osmotic fluid shifts and brain edema. The diagnostic workup should include a history [1,2]. Many medical illnesses, such as congestive heart failure, liver failure, renal failure, and pneumonia, may be associated with hyponatremia.

Hyponatremia is caused either by water retention or (less often) by loss of effective solutes (sodium plus potassium) in excess of water. A decrease in serum sodium concentration creates an osmotic gradient between extracellular and intracellular fluid in cells causing movement of water into cells and consequently cellular edema. Virtually, all the causes of hyponatremia are characterized by an absolute or relative excess of antidiuretic hormone (ADH), most frequently due to the syndrome of inappropriate ADH secretion (SIADH) or to depletion of effective circulating volume, which is a normal stimulus to ADH secretion [3].

The frequency, etiology and clinical association of hyponatremia in patients with acquired immunodeficiency syndrome (AIDS) and AIDS-related complex (ARC) was first reported in 1993 [1]. Hyponatremia in human immunodeficiency disease (HIV) disease and AIDS occurs in 20–80% of hospitalized patients [1], especially in Xinjiang, a developing region in China, where nearly 40% of HIV/AIDS inpatients are hyponatremic. Hyponatremia is the most common electrolyte disorder in clinical practice, and extant evidence indicates that severe hyponatremia is associated with increased morbidity and mortality in HIV/AIDS patients. In a universal model, patients with mild hyponatremia have a 2.0-fold higher risk of death compared with normonatremic patients, while patients with severe/moderate hyponatremia are at 3.4-fold higher risk of death than normonatremic patients [4]. For these reasons, identifying and summarizing the pathogenesis and therapeutic therapies for hyponatremic HIV/AIDS patients are essential and the purpose of the article is to analyze the reasons in the HIV/AIDS patients with hyponatremia and suggest the treatment recommendations.

## 2. The main causes of hyponatremia in HIV/AIDS patients

The World Health Organization (WHO) classifies HIV infection into four stages: Stage 1 (HIV infection), the CD4 + cell count is at least 500 cells per microliter; Stage 2 (HIV infection), the CD4 + cell count is 350–499; Stage 3 (advanced HIV disease), the CD4 + cell count is 200–349; and Stage 4 (AIDS), the CD4 + cell count is less than 200 or the percentage of CD4 + cells is less than 15% of all lymphocytes. Xu reported a significantly positive correlation between serum sodium concentrations and the number of CD4 + cells. It is suggested that the serum sodium level is closely correlated with the severity of patients [4].

**Figure 1.**
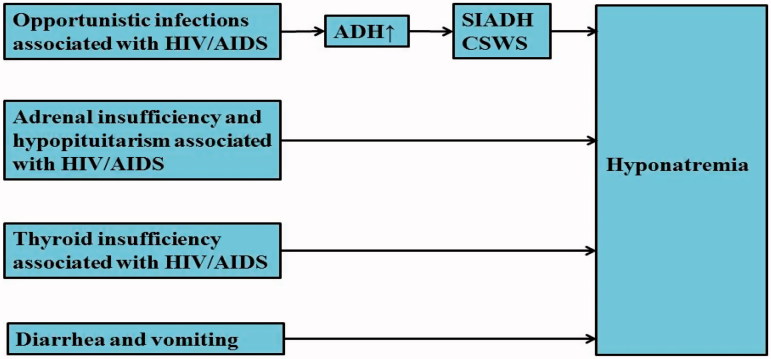
The main causes of hyponatremia in HIV/AIDS patients.

This review focuses on the clinical and pathophysiological aspects of hyponatremia and highlights the causes, presentation and treatment of hyponatremia in HIV/AIDS patients. There are several conditions in patients with HIV/AIDS that may predispose them to the development of hyponatremia: opportunistic infections, adrenal insufficiency and hypopituitarism, thyroid insufficiency, diarrhea and vomiting, etc. [1,3] (Figure 1). More extensive reviews may be beyond the scope of the review.

### 2.1. Hyponatremia due to opportunistic infections in HIV/AIDS

Hyponatremia may not be a frequent side effect of infections, especially in HIV/AIDS patients, and may not cause specific symptoms, which means that it may be overlooked by clinicians.

The most common opportunistic infections in HIV/AIDS patients include bacterial pneumonia, *Mycobacterium tuberculosis* (including tuberculous meningitis [TBM]), *Cryptococcus neoformans*, hepatitis B, hepatitis C and *Plasmodium falciparum* [1]. Indira [5] reported that opportunistic infections should also include oral candidiasis, Cryptococcal meningitis, *Pneumocystis jirovecii* pneumonia, pulmonary tuberculosis and cerebral toxoplasmosis.

Infections of the pulmonary tract and central nervous system (CNS) (such as tuberculous meningitis, encephalitis and abscesses) can induce the release of excess ADH, which is known as the SIADH and cerebral salt wasting syndrome (CSWS).

ADH is synthesized by neuroendocrine neurons in the medial parvocellular part of the paraventricular nucleus in the hypothalamus. It is transported via axons to the neurohypophysis and released into the bloodstream where it acts to increase water absorption at the collecting ducts of the renal tubules. It also has cardiovascular (promotes vasoconstriction) and cerebral effects (reduces cerebral arterial resistance and increases microvascular pressure). Interestingly, ADH also reduces blood flow to the choroid plexus and reduces cerebrospinal fluid (CSF) formation [1]. The osmotic threshold for ADH release is ∼287 mosm/kg; below this concentration, levels of circulating ADH are undetectable. As non-osmotic factors, in particular, hypovolemia, also play a role in the release of ADH, there may be more than one reason for increased ADH levels in patients with meningitis.

Hyponatremia due to SIADH is also a frequent complication of pulmonary infections [6]. However, the underlying mechanisms are uncertain. It has been proposed that a reduction in pulmonary venous return leads to the activation of volume receptors and, consequently, to increased ADH secretion [7]. In accordance with this hypothesis, low-urine sodium has been reported in this clinical context [8].

Moreover, hypoxemia and hypercapnia (usually observed during respiratory infections), alone or in combination, can stimulate the non-osmotic release of ADH [9,10]. CSWS is seldom observed or diagnosed in patients with intracranial disease (most often subarachnoid hemorrhage). However, a variety of infections of the CNS (tuberculous meningitis, poliomyelitis and toxoplasmosis) have also been linked to CSWS [11,12].

SIADH was first described in 1957 by Schwartz et al. [13] in patients with bronchogenic carcinomas [14]. Since then, the condition has been described in association with neurological disorders such as subarachnoid hemorrhage and meningitis, possibly due to hypothalamic injury caused by bleeding or an inflammatory process. SIADH involves the physiologically inappropriate secretion of ADH, or increased renal sensitivity to ADH, leading to renal conservation of water and euvolemic or hypervolemic hyponatremia.

In hyponatremic patients, SIADH diagnosis is based on the presence of normal or reduced urine output, inappropriately concentrated urine, natriuresis, low-serum osmolality, and a normal or slightly raised intravascular volume in the absence of any renal, adrenal or thyroid problems.

A variety of infections of the CNS (encephalitis, tuberculous meningitis, poliomyelitis and toxoplasmosis) have also been linked with CSWS [11,12]. Some studies show that acute kidney injury and hyponatremia are frequent in toxoplasmic encephalitis. Hyponatremia on admission is highly associated with acute kidney injury and mortality [15]. Although CSWS was first described in the 1950 s [16–18], it was subsequently neglected in the literature [19]. Its entrance into the mainstream literature occurred again in 1981 through the work of Nelson et al. [20], who introduced the condition in association with acute neurological disorders. CSWS has been described with a variety of cerebral insults, including TBM and neurosurgical interventions [21].

Berendes et al. showed that although the exact mechanism of CSWS in meningitic disorders is not known, increased levels of atrial natriuretic peptide (ANP) have been described in aneurysmal subarachnoid hemorrhages [22], as has been the case in TBM [23].

The fundamental pathophysiologic mechanisms involved in CSWS are a reduction in sympathetic nervous system outflow during intracranial disease, leading to reduced sodium reabsorption in the proximal tubules, inhibition of the renin-angiotensin-aldosterone system, and release of several natriuretic factors, such as ANP, brain natriuretic peptide (BNP) and other natriuretic proteins [24]. The net effect of these changes is the induction of natriuresis, which, in turn, causes polyuria and a reduction in effective circulating volume, thus leading to hypotension, low central venous pressure (CVP) and hyponatremia. The serum osmolality may be normal or at the low end of normal [24,25].

Essentially, CSWS is characterized by increased loss of urine sodium in combination with extracellular fluid losses due to the accompanying renal water loss. Hyponatremia occurs when the urine sodium loss is greater than the water loss.

Importantly, the misdiagnosis of CSWS as SIADH can be fatal. In CSWS, total body sodium is reduced, whereas total body sodium is normal in SIADH (i.e., the hyponatremia in SIADH is dilutional).

In conclusion, SIADH is caused by excess renal water reabsorption through inappropriate antidiuretic hormone secretion, and fluid restriction is the treatment of choice. On the other hand, cerebral/renal salt wasting syndrome (C/RSW) is caused by natriuresis, which is followed by volume depletion and negative sodium balance; replacement of water and sodium is the mainstay of treatment. Determining the volume status in hyponatremic patients is key for differentiating between SIADH and C/RSW. However, in most situations, differential diagnosis of these two diseases is difficult because they overlap in many clinical and laboratory aspects, especially differences in volume status. Although the distinction between SIADH and C/RSW is difficult, improvement of hypouricemia and increased fractional excretion of uric acid after correction of hyponatremia in SIADH, but not in C/RSW, may facilitate discrimination of the two diseases [26].

### 2.2. Adrenal insufficiency and hypopituitarism in HIV/AIDS patients

Accompanied by opportunistic infections, HIV may disseminate through various organs and result in a variety of complications. However, the dysfunction of specific endocrine glands can easily be overlooked in the absence of a high index of suspicion [27]. Of the endocrine glands, the adrenal gland is the most frequently attacked in HIV/AIDS patients [28].

A morphologic assessment was carried out on the adrenal glands from autopsied HIV/AIDS patients; necrosis, fibrosis, hemorrhages and neoplasias were observed. Inflammatory infiltrates were observed in 99.2% of the cases, with a predominance of mononuclear cells, which affected mainly the medulla, in 97.4% [29]. This inflammation can be explained by the various infectious agents encountered, especially cytomegalovirus (CMV) at the necrosis foci [1]. In addition, the inflammation could be due to the activity of HIV itself, especially medullitis, as the virus has a tropism for the adrenal medulla, which is derived from the neural crista [30] and neural tissue.

Rodrigues et al. suggested that HIV/AIDS and opportunistic infections may contribute to alterations in the adrenal gland that lead to the multiple organ failure observed in terminal AIDS patients [29]. Almost all pathological mechanisms are affected by the adrenal gland, and alterations include deficiencies of cortisol, aldosterone and adrenal androgen. Both cortisol deficiency and aldosterone deficiency contribute to hyponatremia by causing sodium wasting and hypovolemia.

In most patients with hyponatremic hypopituitarism, plasma ADH levels were inappropriately high, probably due to the failure of endogenous cortisol to suppress the hormone in stressful situations. Another main cause of hyponatremia seems to be a failure of endogenous cortisol, which exerts an inhibitory effect on vasopressin secretion [1]. In other words, there is glucocorticoid resistance. There are three mechanisms of glucocorticoid resistance: ligand-induced downregulation of the receptor, dominant-negative inhibition by the beta-isoform of the receptor and repression by the transcription factor NF-kappa B [31]. There are a number of regulatory signaling pathways, such as JNK inhibition [32], PI3K/mTOR inhibition [33], PPARα and fatty acid oxidation [34], etc., which can significantly inhibit glucocorticoid signaling.

### 2.3. Thyroid insufficiency and HIV/AIDS

Among individuals infected with HIV, 1–2% experience overt thyroid disease, and 35% may have subtle abnormalities in thyroid function test findings [1].

In the HIV/AIDS epidemic, viral and opportunistic infections, along with the systemic effects of HIV, have been implicated in many endocrine abnormalities, including those of the thyroid gland. As HIV advances and the immune system no longer functions effectively, a number of opportunistic infections can have both systemic and thyroid-specific effects. According to the literature, infections associated with thyroid dysfunction include *Coccidioides* [35], *Pneumocystis jirovecii* [36], tuberculosis and *Cryptococcus* [37]. These infiltrative conditions can also lead to isolated thyroid abnormalities.

Overt hypothyroidism is defined as the failure of the thyroid to synthesize and secrete adequate T4 into the circulation despite thyroid stimulating hormone (TSH) stimulation. Common symptoms of hypothyroidism include dry skin, cold insensitivity, fatigue, voice changes, constipation and hyponatremia [38].

### 2.4. Hyponatremia due to diarrhea and vomiting

Frequent diarrhea and vomiting induced by HIV/AIDS-related opportunistic infections can lead to hypovolemia via extra-renal salt losses. Diarrhea, defined as loose stools, occurs when the intestine does not complete absorption of electrolytes and water from luminal contents. This can occur when a nonabsorbable, osmotically active substance is ingested (“osmotic diarrhea”) or when electrolyte absorption is impaired (“secretory diarrhea”). Most cases of acute and chronic diarrhea are due to the latter mechanism. Secretory diarrhea can result from bacterial toxins, reduced absorptive surface area caused by disease or resection, luminal secretagogues (such as bile acids or laxatives), circulating secretagogues (such as various hormones, drugs and poisons), and medical problems that compromise regulation of intestinal function [39].

HIV-infected individuals suffer from enteropathy during the acute phase of the infection through the advanced stages of the disease. It involves diarrhea, increased gastrointestinal inflammation, malabsorption of bile acids and vitamin B12, and increased intestinal permeability (up to five-fold higher than in healthy controls) [40].

It has also been suggested that HIV has a direct “virotoxic” effect on enterocytes in the early stages of infection. HIV infection directly causes dramatic damage to the gastrointestinal tract (GIT) that includes substantial disruption of gut microbiota composition with an increased prevalence of pathogenic microbes and a reduced prevalence of less-aggressive indigenous organisms, massive loss of gut-residing CD4^+^ T cells and downregulation of GIT gene expression [41].

## 3. Our treatment experience and recommendations

We agree with Menon that the management of hyponatremia due to HIV-specific causes such as drugs (including antiretrovirals), endocrine syndromes and salt wasting appears to require the same general treatment principles as in non-HIV cases [42]. Our treatment experience and recommendations are described below (Table 1). Of course, these matters remain open for discussion.

**Table 1.** Treatment and the effectiveness of treatment of various diseases in HIV/AIDS patients.

Disease	Treatment	Effectiveness
SIADH and CSWS	0.9% or 3% Sodium chloride, ≥0.5 mmol/l/h Mineralocorticoid	Safe and effective treatment
Adrenal insufficiency and hypopituitarism	Hydrocortisone (30–60 mg per day, beginning 1–5 days after admission)	Serum sodium usually returns to normal within 3–5 days
Thyroid insufficiency	Levothyroxine	TSH levels return to normal in 6–8 weeks
Diarrhea and vomiting	Probiotic bacteria	Provide specific benefits in HIV-1 infection

### 3.1. For opportunistic HIV/AIDS infections

Aside from the treatment of the opportunistic infection, volume replacement achieved with 0.9% (or 3% sodium chloride if necessary), is the major treatment for SIADH and CSWS. The rapidity of salt replacement depends on the rate at which the hyponatremia developed. Treatment of hyponatremia developing at a rate of ≥0.5 mmol/l/h should be aggressive, as it is a life-threatening complication and may cause death from severe cerebral edema and cerebral herniation [43,44]. Readers can also refer to the Adrogue–Maddias formulae [45], which is used widely. In addition, fluid restriction is an effective treatment for SIADH because the underlying problem is inappropriate water retention. Demeclocycline, a vasopressin antagonist, is used to treat SIADH, but its effects may be unpredictable.

According to the review by Elhassan and Schrier, the introduction of vaptans has been a major advancement in the treatment of hyponatremia. Vaptans include specific non-peptide V2 receptor antagonists (tolvaptan, lixivaptan and satavaptan) and a dual V1/V2 receptor antagonist (conivaptan). These medications act by increasing electrolyte-free water excretion without appreciable effects on urine sodium or potassium excretion. However, among these agents, only tolvaptan and conivaptan are approved by the United States Food and Drug Administration (FDA), and can be initiated only in a hospital setting where serum sodium can be monitored closely due to the risk of overly rapid correction of hyponatremia [46].

Despite its effectiveness for SIADH, volume restriction is detrimental to patients with CSWS [44], and it can cause a further reduction in cerebral perfusion pressure. Because ANP can inhibit mineralocorticoid secretion in patients with CSWS, administration of an agent with mineralocorticoid activity, such as fludrocortisone, has been shown to effectively return serum sodium levels to normal [47] by acting directly on the renal distal tubules to enhance sodium reabsorption. In our patients, mineralocorticoid supplementation seemed to be a safe and effective treatment for CSWS, whereas normal saline and hypertonic saline may be temporary measures.

### 3.2. For adrenal insufficiency and hypopituitarism in HIV/AIDS patients

In our patients, mineralocorticoid supplementation seemed to be a safe and effective treatment for hyponatremia due to adrenal insufficiency and hypopituitarism, whereas normal saline and hypertonic saline may be temporary measures. Because the number of patients is very limited, there is no clear timetable for hyponatremia due to adrenal insufficiency and hypopituitarism. Patients in our hospital recovered after low-dose hydrocortisone substitution. All patients were given oral or intravenous hydrocortisone (30–60 mg a day), beginning 1–5 days after admission, which usually led to a return of serum sodium to normal within 3–5 days, but some patients need a longer course of treatment.

### 3.3. For thyroid insufficiency in HIV/AIDS patients

Levothyroxine should be used to treat hypothyroidism and to maintain TSH levels within normal reference limits. Care must be taken when patients are receiving highly active antiretroviral therapy (HAART) because drug interactions have been reported, especially with protease inhibitors, which may share the glucuronidation metabolic pathway [48,49]. TSH levels should be monitored 6–8 weeks after therapy initiation to determine whether levels have returned to normal. If TSH levels are still too low, the dose should be adjusted accordingly. Current guidelines do not support the combined use of levothyroxine and triiodothyronine (T3) or the use of desiccated thyroid hormones [50].

### 3.4. For diarrhea and vomiting

Current and emerging research supports the concept that probiotic bacteria can provide specific benefits in HIV-1 infection. Probiotic bacteria have proven active against bacterial vaginosis in HIV-1-positive women and have enhanced growth in infants with congenital HIV-1 infections [1].

## 4. Conclusions

Hyponatremia is a common manifestation of HIV/AIDS opportunistic infections. These infections result in increased ADH release and can lead to the development of both SIADH and CSWS. In addition, opportunistic infections, as well as HIV itself, can lead to the dysfunction of many endocrine organs, including insufficiencies in the adrenal gland and thyroid glands and hypopituitarism. Hyponatremia is associated with increased morbidity and mortality in HIV/AIDS, and it remains challenging for physicians to identify effective diagnostic criteria and treatments.
