# Correction to: Shu, et al., HIV/AIDS-related hyponatremia: an old but still serious problem

**DOI:** 10.1080/0886022X.2018.1440784

**Published:** 2018-02-20

Shu Z, Tian Z, Chen J, Ma J, Abudureyimu A, Qian Q, Zhuo L. HIV/AIDS-related hyponatremia: an old but still serious problem. *Renal Failure*. 2018;40:68–74. https://doi.org/10.1080/0886022X.2017.1419975.

When the above article was published online, Halmurat Upur was listed as the second author. However, he considers that he had contributed too little to the article, so he has requested to remove his name from the author list.

The authors apologize for this error.

